# Pharmacological inhibition of the cysteine protease cathepsin C improves graft function after heart transplantation in rats

**DOI:** 10.1186/s12967-023-04659-6

**Published:** 2023-11-09

**Authors:** Baoer Liu, Brice Korkmaz, Patricia Kraft, Tobias Mayer, Alex A. Sayour, Marc A. Grundl, Roxane Domain, Matthias Karck, Gábor Szabó, Sevil Korkmaz-Icöz

**Affiliations:** 1 Department of Cardiac Surgery, University Hospital Heidelberg, 69120 Heidelberg, Germany; 2 Department of Cardiac Surgery, University Hospital Halle (Saale), 06120 Halle, Germany; 3 INSERM UMR-1100, “Research Center for Respiratory Diseases” and Université de Tours, 37032 Tours, France; 4 Heart and Vascular Center, Semmelweis University, Budapest, 1122 Hungary; 5 Department of Medicinal Chemistry, Boehringer Ingelheim Pharma GmbH & Co KG, 88397 Biberach a.d. Riss, Germany

**Keywords:** Heart transplantation, Ischemia, Reperfusion injury, Cathepsin C, Neutrophil elastase

## Abstract

**Background:**

Heart transplantation (HTX) is the standard treatment for end-stage heart failure. However, reperfusion following an ischemic period can contribute to myocardial injury. Neutrophil infiltration, along with the subsequent release of tissue-degrading neutrophil elastase (NE)-related serine proteases and oxygen-derived radicals, is associated with adverse graft outcomes. The inhibition of cathepsin C (CatC) has been shown to block NE-related protease activation. We hypothesized that the CatC inhibitor BI-9740 improves graft function after HTX.

**Methods:**

In a rat model of HTX, the recipient Lewis rats were orally administered with either a placebo (n = 12) or BI-9740 (n = 11, 20 mg/kg) once daily for 12 days. Donor hearts from untreated Lewis rats were explanted, preserved in a cardioplegic solution, and subsequently heterotopically implanted. In vivo left-ventricular (LV) graft function was assessed after 1 h of reperfusion. The proteolytic activity of neutrophil serine proteases was determined in bone marrow lysates from BI-9740-treated and control rats. Additionally, myocardial morphological changes were examined, and heart samples underwent immunohistochemistry and western blot analysis.

**Results:**

The NE-related proteolytic activity in bone marrow cell lysates was markedly decreased in the BI-9740-treated rats compared to those of the placebo group. Histopathological lesions, elevated CatC and myeloperoxidase-positive cell infiltration, and nitrotyrosine immunoreactivity with an increased number of poly(ADP-ribose) polymerase (PARP)-1-positive cells were lowered in the hearts of animals treated with BI-9740 compared to placebo groups. Regarding the functional parameters of the implanted graft, improvements were observed in both systolic function (LV systolic pressure 110 ± 6 vs 74 ± 6 mmHg; dP/dt_max_ 2782 ± 149 vs 2076 ± 167 mmHg/s, LV developed pressure, at an intraventricular volume of 200 µl, *p* < 0.05) and diastolic function in the hearts of BI-9740 treated animals compared with those receiving the only placebo. Furthermore, the administration of BI-9740 resulted in a shorter graft re-beating time compared to the placebo group. However, this study did not provide evidence of DNA fragmentation, the generation of both superoxide anions and hydrogen peroxide, correlating with the absence of protein alterations related to apoptosis, as evidenced by western blot in grafts after HTX.

**Conclusions:**

We provided experimental evidence that pharmacological inhibition of CatC improves graft function following HTX in rats.

**Supplementary Information:**

The online version contains supplementary material available at 10.1186/s12967-023-04659-6.

## Background

Heart transplantation (HTX) is recognized as an optimal therapy for patients with end-stage heart diseases. However, ischemia/reperfusion (IR) injury during HTX is inevitable and associated with postoperative complications, such as graft dysfunction and reduced graft survival [1, 2]. Researchers have devoted significant effort to understand the underlying cellular and molecular mechanisms governing IR injury. Intracellular calcium accumulation, cardiac metabolic alterations, generation of reactive oxygen species (ROS), production of inflammatory cytokines, and activation of inflammatory cells have been suggested as major contributors to myocardial IR injury [3–7]. Besides macrophages, especially neutrophils [8], have been hypothesized to contribute to reperfusion injury by acting as early responders to inflammatory stimuli. They increase proteolytic activity at the site of inflammation via release of pro-inflammatory proteases, in addition to increasing ROS activity.

Pro-inflammatory neutrophil serine proteases (NSPs), including neutrophil elastase (NE), proteinase 3, cathepsin G (CatG), and the recently discovered NSP4, are initially synthesized as inactive zymogens [9, 10] that are converted into their mature active form by cathepsin C (CatC, also known as dipeptidyl peptidase I (DPPI)) within neutrophil progenitor cells before the mature neutrophils are released from the bone marrow into circulation [11, 12]. Under physiological conditions, these mature NSPs play important roles in both intracellular and extracellular bacteria killing, as well as the regulation of inflammatory processes [13, 14]. However, under pathological conditions, data indicates they can contribute to chronic inflammatory diseases, auto-immune diseases, and cancer [9, 10, 15], when massive release of active NSPs is not counterbalanced and regulated by the presence of endogenous serine protease inhibitors [10]. In order to inhibit the activation of these NSPs during neutrophil maturation, hence reducing the overall release of proteolytically active NSPs at the site of inflammation and re-establishing a balanced protease-antiprotease environment, CatC inhibitors have been investigated. As a result, several small molecule inhibitors of CatC are currently undergoing preclinical and clinical trials to evaluate their efficacy in several indications [9, 15, 16]. We investigated a novel concept in our rat model of heterotopic HTX by targeting the uncontrolled NSPs through direct inhibition of their activation via the enzyme CatC [10]. This study aimed to explore the impact of NSPs' activity on IR injury.

Based on these findings, we hypothesized that administering the novel nitrile CatC inhibitor, BI-9740, to the heart transplant recipients improves left-ventricular (LV) graft function in a rat model of HTX.

## Methods

### Animals

Male Lewis rats weighing 250–300 g (Janvier Labs, Saint Berthevin, France) were housed at a constant temperature of 20–24 ℃ with a 12-h of light/dark cycles and were acclimatized for at least 1 week. Standard laboratory rodent diet and water were supplied ad libitum. All animals received human care in consistency with the “Principles of Laboratory Animal Care” defined by the National Society for Medical Research, as well as the “Guide for the Care and Use of Laboratory Animals” arranged by the Institute of Laboratory Animal Resources and distributed by the National Institutes of Health (NIH Publication, 8th Edition, 2011). Approval for animal experimentation was granted by the Ethical Committee of the Regional Council of Karlsruhe, Germany (G257/20).

### Preparation of CatC inhibitor BI-9740

The small molecule CatC inhibitor BI-9740, obtained for free via Boehringer-Ingelheim Pharma’s (Ingelheim am Rhein, Germany) open innovation portal OpnMe (https://opnme.com), was dissolved in a solution containing 0.5% Natrosol^™^ 250 HX Pharm (Pharmacy of the University Hospital Heidelberg, Germany) and 0.015% Tween-80 (Merck KGaA, Darmstadt, Germany). This vehicle solution was generated and employed in line with the standard protocol used for pharmacodynamics and pharmacokinetic studies with BI-9740 at Boehringer-Ingelheim. Briefly, the vehicle solution was added to the weighted BI-9740 compound and vortexed. Gradually, hydrochloric acid was introduced to adjust the pH value to 4.0 while stirring. This mixture was vortexed and sonicated until a clear solution was obtained and stored at room temperature in the dark for a maximum of 3 days. The concentration of the stock solutions was 2 mg/ml.

In mice, BI-9740 inhibits CatC with an IC_50_ (half-maximal inhibitory concentration) of 0.6 nM, while rats exhibited a value of 2.6 nM (~ fourfold difference). Mice displayed a plasma protein binding rate of 97.7%, whereas rats showed 99.9%, a difference of ~ 20 fold regarding the unbound concentration of BI-9740. In an efficacy model in mice, administration of BI-9740 led to a reduction of NE activity by 91%, proteinase 3 by 97%, and CatG by 100% at a dosage of 0.5 mg/kg. Considering this in vivo activity in mice, the very similar reported oral bioavailability of BI-9740 in mouse and rats, as well as the aforementioned species difference in potency of fourfold between and a difference of 20 fold in free, not plasma bound concentration, we in first approximation assumed that a dose of 20 mg/kg in rats should be sufficient to lead to a significant level of CatC and hence NSP inhibition in rats comparable to the 0.5 mg/kg does in mice. Detailed data on the structure, potency, and plasma protein binding is available at https://opnme.com/molecules/cathepsin-c-inhibitor-bi-9740.

### Heterotopic HTX model

#### Experimental groups

The animals were randomly divided into three groups. The recipient rats were administered with either BI-9740 (HTX + BI-9740 group, n = 11; 20 mg/kg body weight and administration volume: 1 ml/100 g body weight) or an equal volume of 0.5% Natrosol^™^ 250 HX Pharm vehicle (HTX group, n = 12) once daily for 12 days prior to transplantation. In the control group (n = 10), rats neither received treatment nor underwent transplantation.

#### Heterotopic HTX in rats

The model was established as previously described [17]. Briefly, donor Lewis rats were anesthetized with 3% isoflurane gas in a chamber, and maintained with inhalation from a connected tube with 1.75–2.5% in O_2_, and heparinized (400 IU/kg). Custodiol solution (Dr. Franz Köhler, Chemie GmbH, Bensheim, Germany) was used to induce cardiac arrest and to preserve the explanted heart at 4 °C. On day 13, recipient Lewis rats were anesthetized and heparinized, as described above. The donor heart’s aorta was anastomosed to the recipient’s abdominal aorta, and the pulmonary artery was anastomosed to the inferior vena cava. The duration of cold ischemia is standardized to 60 min, including both explantation and preservation time, followed by 30 min of implantation. Blood reperfusion was standardized to 60 min and the re-beating time (time to restoration of heartbeat) was also recorded. Additionally, the graft survival rate (i.e. a functioning graft at the time of cardiac function measurement) was calculated.

#### Post-transplant graft function measurements

Graft function was measured in vivo 60 min after reperfusion, as we previously described [17]. A 3F balloon catheter (Dispomedica, Hamburg, Germany) and a 2F Millar microprobe pressure catheter (SPR-320NR, Millar Instruments, Houston, TX, USA) were inserted into the left ventricle via the apex. The intraventricular volume was determined using the volume of the deflated balloon (0.02 ml) plus the volume of saline injected into the balloon, with 0.03 ml added each time from 0 to 0.18 ml, resulting in a gradual increase of the final intraventricular volume from 0.02 to 0.20 ml. LV systolic pressure (LVSP), the maximal rate of rise of LV pressure (dP/dt_max_) and the maximum rate of fall of LV pressure (dP/dt_min_) were measured at different LV volumes. Following the hemodynamic measurements, all rats were humanely sacrificed under anesthesia by bleeding. Subsequently, the transplanted hearts were excised for further analysis. At the end of the study, the rat cadavers were placed in a −20 °C freezer for preservation and later transferred to the designated animal carcass processing facility.

### Measurement of selective NE and CatG proteolytic activities

Rat NE and CatG activities in bone marrow lysates from BI-9740-treated and control rats were measured spectrofluorometrically (Ex: 320 nm and Em: 420 nm) using Abz-peptidyl-EDDnp fluorescence resonance energy transfer substrates, as described previously [18]. Briefly, bone-marrow cells were lysed on ice and the soluble fractions were separated from cell debris by centrifugation. NE activity (10 µg of total proteins) was measured with or without human alpha-1 antitrypsin (Sigma-Aldrich, SRP6312) (5 µM, 1 h at 37 °C, in HEPES 50 mM, NaCl 0.75 M, NP40 0.05%, pH 7.4) using Abz-APQQIMDDQ-EDDnp (20 µM) as substrate. CatG activity (0.5 µg of total proteins) was measured with or without human AAT (5 µM, 1 h at 37 °C, in HEPES 50 mM, NaCl 100 mM, NP40 0.05%, pH 7.4) using Abz-TPFSGQ-EDDnp (20 µM) as substrate.

### Histological process

Upon completion of functional measurements, the transplanted heart samples were immediately explanted, fixed in a buffered paraformaldehyde solution (4%), embedded in paraffin, and cut using a microtome. Then, 4-µm thick sections were placed onto adhesive slides. These samples were stained with hematoxylin and eosin (HE) for histopathological examination. Immunohistochemical staining was performed for nitrotyrosine (1:1000; Ab7048, Abcam, Berlin, Germany), MPO (1:100, Abcam, Berlin, Germany), poly(ADP-ribose) polymerase (PARP)-1 (1:1000, Abcam, Berlin, Germany) and CatC (1:300, Santa Cruz Biotechnology, Inc., Heidelberg, Germany). The presence of ROS was determined in situ using the oxidative fluorescent dye dihydroethidium [19]. To detect DNA strand breaks, the Terminal deoxynucleotidyl transferase-mediated dUTP nick-end labeling (TUNEL) assay was performed, as we previously described [20].

### Quantification of immunohistochemical staining

HE staining was used to examine the morphology of myocardial tissue. The number of MPO-, CatC-, PARP-1, and TUNEL-positive cells were counted. The quantification of MPO-positive cells was based on the total numbers of MPO-positive cells, whereas CatC-, PARP-1, and TUNEL-positive cells were expressed as a percentage over the total cell count. Quantitative evaluation was performed using Image J software (NIH, Maryland, USA). A semi-quantitative evaluation of nitrotyrosine expression was conducted by assessing both the staining intensity (with 0 indicating absolute negativity, 1 for low positivity, 2 for medium positivity and 3 for high positivity) and the number of the labeled target protein (with 0 for negative, 1 for 1–5, 2 for 6–10, 3 for 11–15, 4 for greater than 15). The total score for each field was calculated by multiplying the intensity score by the area score (0–12). The fluorescence intensity score (ranging from 0 to 3) within myocardial sections stained with dihydroethidium was visualized using a fluorescence microscope (Olympus Model BX51, Japan). The evaluation was performed in a blinded manner, with randomly selected non-overlapping fields per myocardium per rat.

### Sodium dodecyl sulfate (SDS)—polyacrylamide gel electrophoresis (PAGE) and immunoblotting analysis

Bone marrow lysates were separated on a 12% SDS-PAGE in reducing/denaturing conditions (50 μg of protein/lane) and blotted onto nitrocellulose membranes. The membranes were then saturated with phosphate-buffered saline (PBS), 0.1% Tween, 5% nonfat milk and incubated with NE-antibody (1:800 diluted in PBS, 0.1% Tween, 5% nonfat milk) overnight at 4 °C. Following this, the membranes were washed 3 times in PBS, 0.1% Tween and then incubated for 1.5 h with a peroxidasecoupled secondary antibody (anti-rabbit IgG-horseradish peroxidase, Jackson ImmunoResearch, 111-035-003) diluted 1:5000 in PBS, 0.1% Tween and 5% nonfat milk. The membranes were washed again as previously described, and the detection was performed using the Enhanced Chemiluminescence system (Cytiva, RPN2232). The same membranes were stripped using hydrogen peroxide 35% for 20 min at 37 °C and immunoblotting was performed using an anti-myeloperoxidase antibody (MPO) (Santa Cruz, sc-16128-R) (1:1000 diluted in PBS, 0.1% Tween, 5% nonfat milk) in the same conditions as before. Densitometry was performed using ImageJ software and the NE/MPO ratio was analyzed.

Proteins were extracted from the myocardial tissue in RIPA buffer (Melford, Ipswich, UK). The protein concentration was determined using the Pierce^™^ BCA Protein Assay Kit (Thermo Fisher Scientific, Karlsruhe, Germany). Total protein homogenates 20 µg/30 µl were denatured, separated on SDS-PAGE electrophoresis gels, and subsequently transferred onto a polyvinylidene fluoride membrane (Millipore, Darmstadt, Germany). The membrane was blocked with 5% bovine serum albumin (BSA) in Tris-buffered saline (TBS) containing Tween 20 for 1 h, followed by overnight incubation at 4 °C with caspase-3 antibody (1:1000 in 2,5% BSA/TBS-Tween, abcam Cambridge, UK), bcl-2 (1:1000 in 2,5% BSA/TBS-Tween, abcam Cambridge, UK) and bax (1:1000 in 2,5% BSA/TBS-Tween, abcam Cambridge, UK). After washing the blots to remove excessive binding of the primary antibody, the blots were incubated for 1 h with a horseradish peroxidase-conjugated secondary antibody at room temperature. Glyceraldehyde-3-phosphate dehydrogenase (GAPDH) and beta-actin, housekeeping proteins, were used as loading controls and for protein normalization. The immunoreactive protein bands were developed using the Enhanced Chemiluminescence system (PerkinElmer, Rodgau-Juegesheim, Germany). The intensity of the immunoblot bands was detected using a Chemismart 5100.

### Statistical analysis

Data are expressed as mean ± standard error of the mean (SEM). Statistical analyses were conducted using SPSS version 24 (IBM SPSS Statistics, New York, USA). The normality of data distribution was assessed using the Shapiro–Wilk normality test. In cases where the data exhibited a normal distribution, the two-sample student t-test was employed to examine the differences between the two groups. If the assumption of normality was not met, a nonparametric Mann–Whitney test was utilized. For graft function measurements, including LVSP, developed pressure, dP/dt_max_, rate pressure product, and dP/dt_min_, a two-factor mixed analysis of variance (ANOVA) and Tukey’s post-hoc test were carried out for multiple comparisons. To detect differences among the three experimental groups, a one-way ANOVA and Tukey’s post-hoc-test were conducted. If the data did not pass the normality test, the nonparametric Kruskal–Wallis test followed by Dunn’s post-hoc-test was used. A p < 0.05 was considered statistically significant.

## Results

### Effect of prolonged BI-9740 administration on body weight and NSP activity

**Fig. 1:**
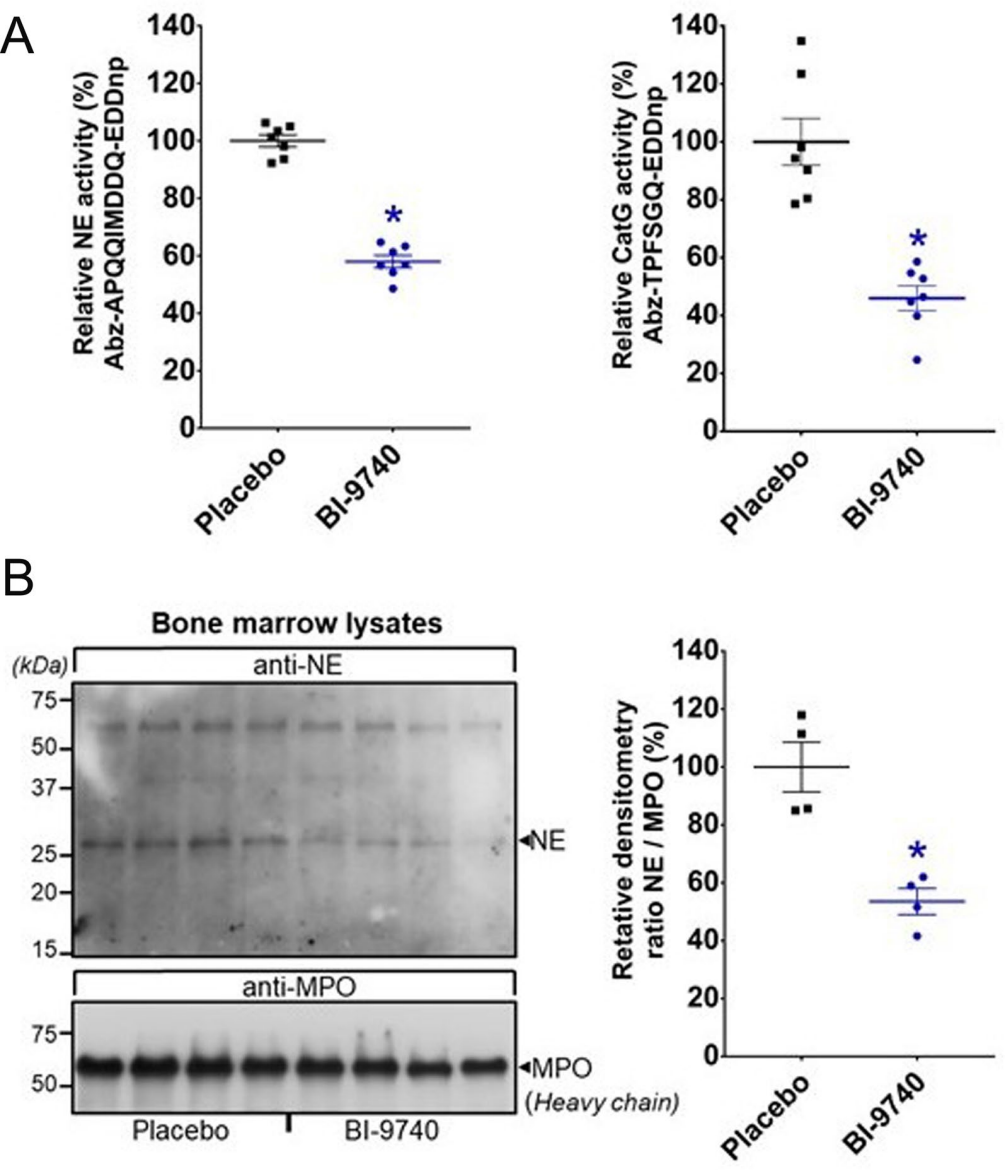
Effect of BI-9740 on bone marrow neutrophil serine protease activation. **A** Activities of neutrophil elastase (NE) and cathepsin G (CatG) and **B** Western blotting analysis of NE in bone marrows from placebo and BI-9740-treated rats. The upper blot was incubated with anti-NE, and the lower blot was incubated with anti-myeloperoxidase (MPO) for use as the loading control. Values are presented as mean ± SEM. *p < 0.05 vs. placebo

Oral administration of BI-9740 at a dose of 20 mg/kg body weight for a duration of 12 days did not have any effect on the body weight when compared to the placebo-treated animals (312 ± 3.3 g vs 311 ± 5 g, *p* > 0.05). As the pharmacological blockade of CatC should occur in the bone marrow, blood and bone-marrow exposure to BI-9740 after 12 days of treatment was assessed. The proteolytic activity of two major NSPs was determined in bone marrow lysates from BI-9740- and placebo-treated rats. Our results showed that BI-9740 treatment significantly reduced the activity of NE and CatG (by 42 ± 2% for NE, 54 ± 4% for CatG, all *p* < 0.0001) in comparison to the placebo group (Fig. 1A). Furthermore, Western blotting showed reduced NE protein (by 46 ± 5%, p = 0.0015) in the BI-9740-treated rats compared to the placebo rats (Fig. 1B). Thus, 12 days of BI-9740 administration were well-tolerated without obvious adverse events, did not induce any wasting, and significantly reduced bone-marrow NSP proteolytic activities.

### Effect of BI-9740 administration on myocardial morphology after HTX

**Fig. 2:**
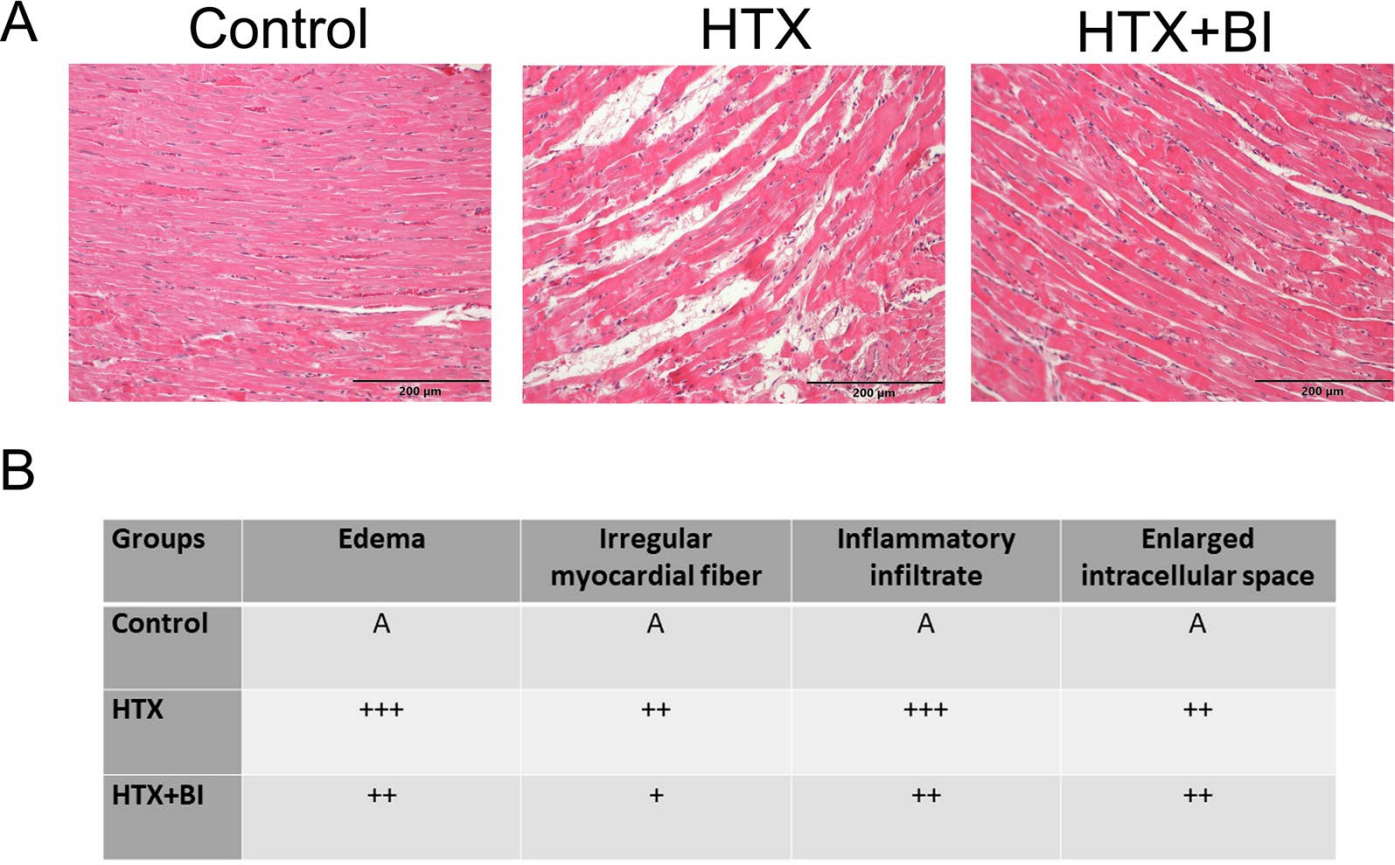
Effect of BI-9740 (BI) on myocardial morphology in grafts after heart transplantation (HTX). **A** Representative images of hematoxylin and eosin (HE) staining (× 200, scale bar: 200 µm) on cardiac tissue samples from the control group (41 pictures from 7 rats), HTX group (66 pictures from 11 rats) and HTX + BI group (44 pictures from 8 rats). In the HE staining images, nuclei are stained blue, cytoplasm is stained red, and collagen fibers display varying shades of red. **B** The degree of histopathological changes was assessed by examiners who were blinded to the experimental groups. The evaluation was conducted across 3–8 randomly selected, non-overlapping fields within each rat’s myocardial tissue. A indicates no changes; + , mild changes; +  + , moderate changes; and +  +  + , marked changes

In the HE staining images, nuclei are stained blue, cytoplasm is stained red, and collagen fibers display varying shades of red. In the control group, the morphology of the myocardial tissue was normal (Fig. 2A). Cardiomyocytes are orderly and closely arranged, with a small intercellular space, and no edema is present between cells (Fig. 2A). In contrast, the HTX group showed disorganization of myofibers, which is related to a dense infiltration of inflammatory cells and interstitial edema (Fig. 2A). BI-9740 treatment exhibited reduced irregularly arranged fiber, decreased inflammatory infiltrate, and less edema in the grafts after HTX (Fig. 2A). Figure 2B illustrates the effect of BI-9740 on the extent of histological changes in myocardial tissues.

### Effect of BI-9740 on CatC, MPO, nitrotyrosine and PARP-1 immunoreactivity after HTX

**Fig. 3:**
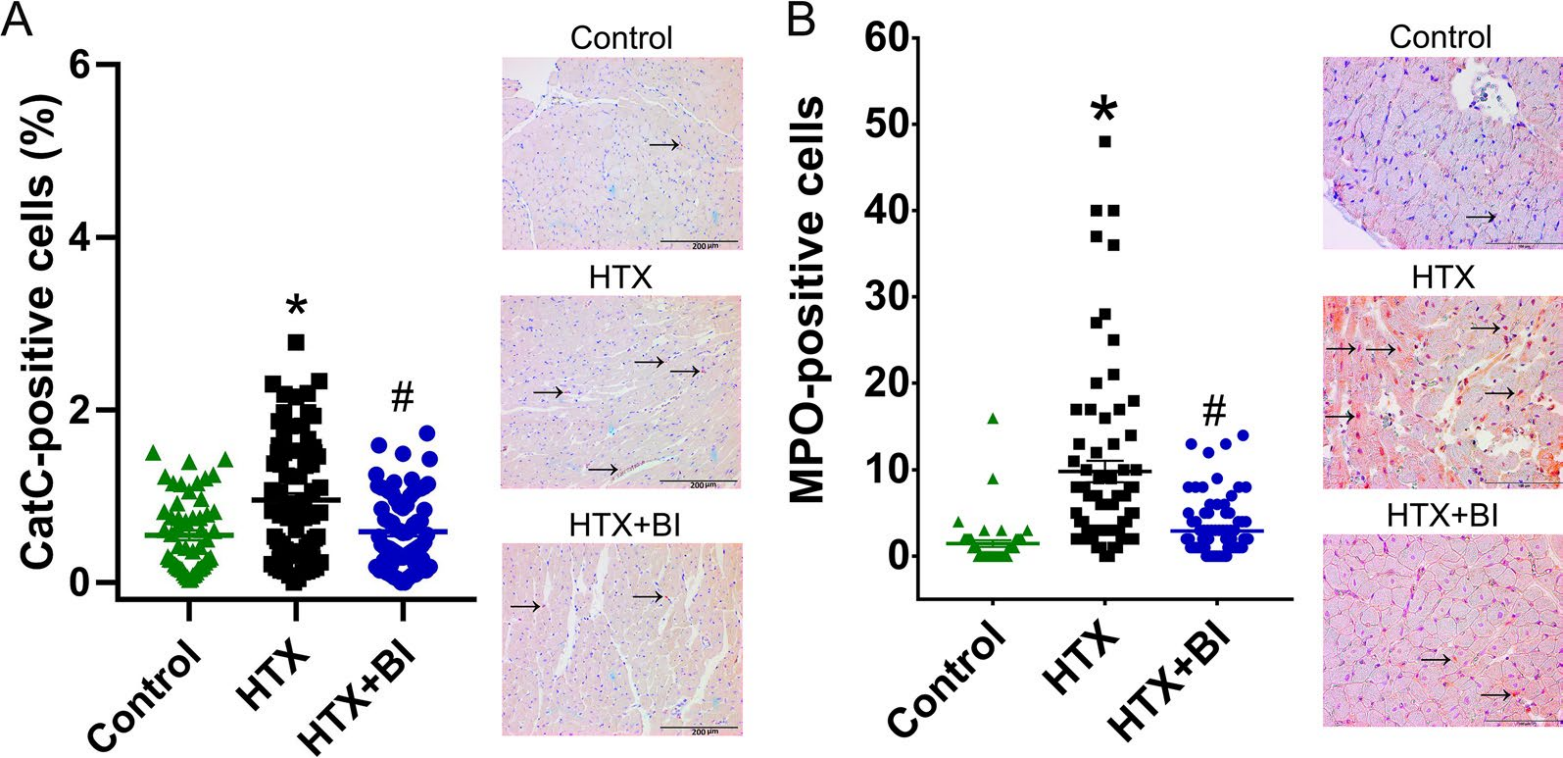
Effect of BI-9740 (BI) on cathepsin C (CatC) and myeloperoxidase (MPO) expression in grafts after heart transplantation (HTX). Representative images of **A** CatC (× 200, scale bar: 200 µm) and **B** MPO (× 400, scale bar: 100 µm) immunostaining followed by quantification based on the percentage of CatC-positive cells over the total cell count (control: 56 pictures from 7 rats; HTX: 80 pictures from 10 rats; HTX + BI: 76 pictures from 10 rats) and the total number of MPO-positive cells in the tissue section (control: 47 pictures from 6 rats; HTX: 72 pictures from 9 rats; HTX + BI: 88 pictures from 11 rats). The samples were assessed by two examiners who were blinded to the experimental groups. The evaluation was carried out on 4–8 randomly selected non-overlapping fields within each rat’s myocardial tissue. Values are expressed as mean ± SEM and correspond to the mean of all pictures. *p < 0.05 vs. control, ^#^*p* < 0.05 vs HTX

**Fig. 4:**
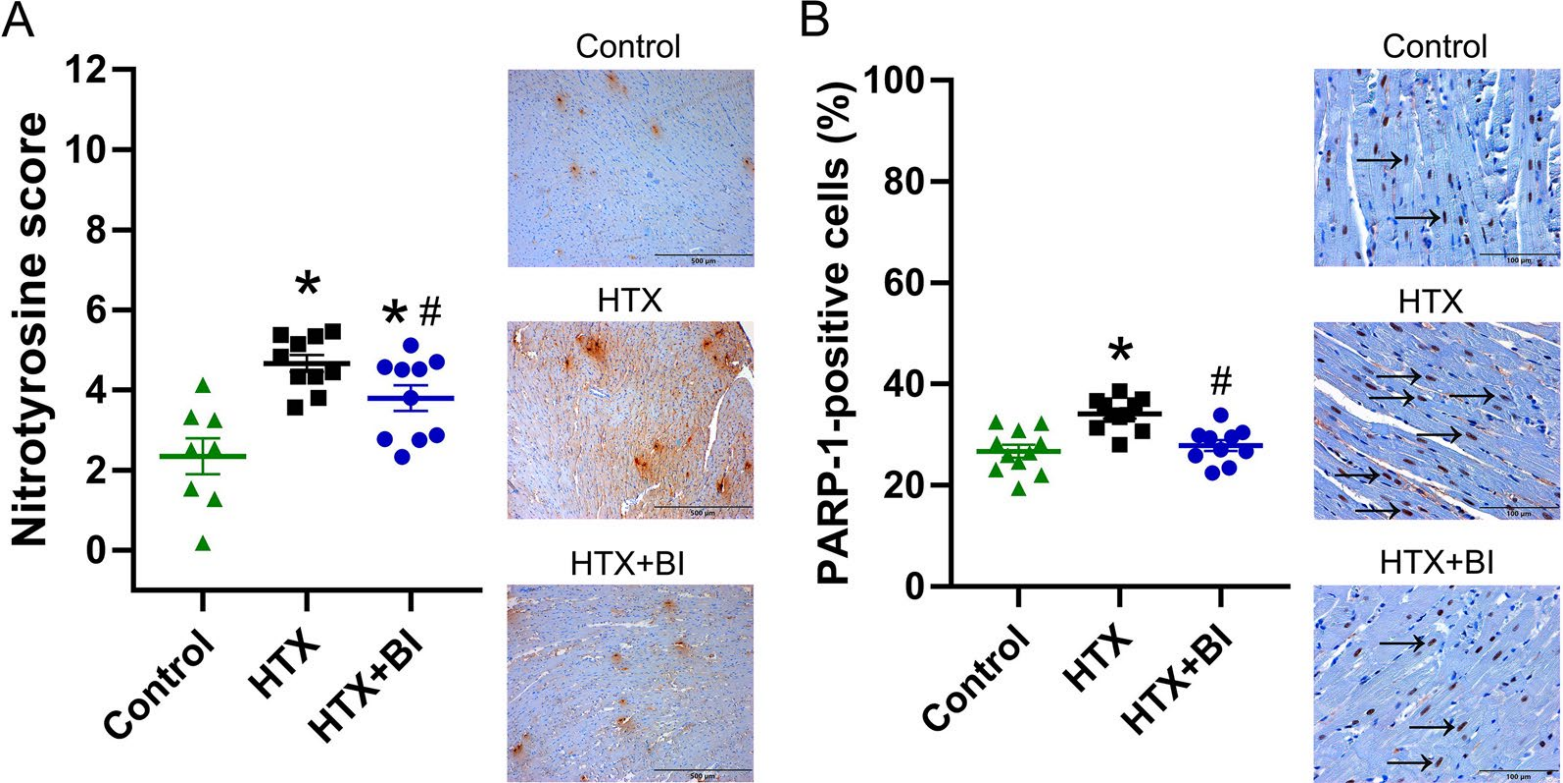
Effect of BI-9740 (BI) on nitrotyrosine and poly(ADP-ribose) polymerase (PARP)-1 expression in grafts after heart transplantation (HTX). Representative images of **A** nitrotyrosine (× 100, scale bar: 500 µm) and **B** PARP-1 (× 400, scale bar: 100 µm) immunostaining are shown, along with semi-quantitative analysis of nitrotyrosine immunoreactivity (control: 8 rats, HTX: 10 rats, HTX + BI: 10 rats) and quantification of PARP-1-positive cells (control: 10 rats, HTX: 11 rats, HTX + BI: 10 rats). The samples were assessed by two examiners who were blinded to the experimental groups. The evaluation was conducted on 4–8 randomly selected non-overlapping fields per rat’s myocardial tissue. Values are expressed as mean ± SEM. *p < 0.05 vs. control, ^#^*p* < 0.05 vs HTX

Immunohistochemical analysis revealed an increase in the number of myocardial CatC-positive cells after HTX compared to control hearts. This increase was significantly decreased by BI-9740 treatment (Fig. 3A). When the cells were counted, an elevation in the number of MPO-positive cells was found in the graft after HTX, indicating infiltrating neutrophils, which was significantly decreased by BI-9740 administration (Fig. 3B). Additionally, the increased immunoreactivity of nitrotyrosine, a biomarker of peroxynitrite, and the increased PARP-1-positive cells, an indicator of apoptotic responses, in the HTX hearts were significantly attenuated by BI-9740 administration (Fig. 4A, B). Therefore, 12 days of BI-9740 treatment was shown to decrease myocardial neutrophil infiltration and nitro-oxidative stress with a decrease in the number of PARP-1-positive cells.

### Effect of BI-9740 on ROS generation and DNA strand breaks after HTX

We have performed dihydroethidium staining to identify ROS generation and TUNEL assay to detect DNA strand breaks. The generation of both superoxide anion and hydrogen peroxide was found to be similar among all the experimental groups (Additional file 1: Figure S1A). Furthermore, quantification of TUNEL-positive nuclei revealed no significant difference among the groups (Additional file 1: Figure S1B).

### Effect of BI-9740 on protein expression of Bax, Bcl-2 and caspase-3 after HTX

**Fig. 5:**
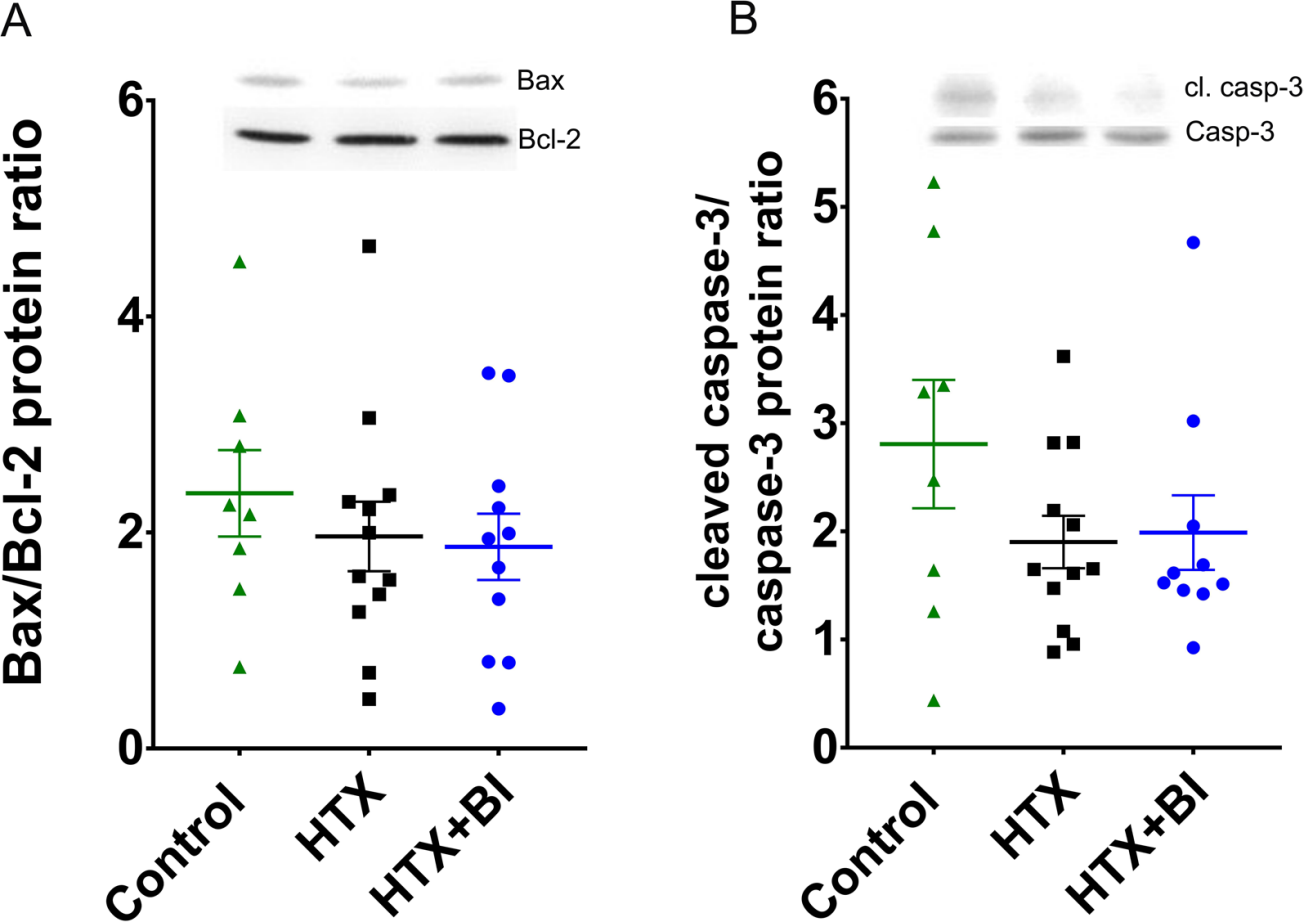
Effect of BI-9740 on protein expression of bcl-2, bax, and caspase-3 (casp-3) in grafts after heart transplantation (HTX). Quantification of the protein bands showing **A** bax-to-bcl-2 ratio, and **B** cleaved (cl.) caspase-3-to-caspase-3 ratio. Values are expressed as mean ± SEM

Since oxidative stress induces apoptosis, we evaluated the effect of BI-9740 on Bax and Bcl-2 levels, as well as procaspase-3 cleavage, which serves as an indicator of pro-caspase-3 activation. Consistent with the results from ROS generation and TUNEL assay experiments, our Western blot and densitometric analysis of these tested proteins did not reveal significant differences among the groups (Fig. 5).

### Effect of BI-9740 on heart rate and re-beating time after HTX

Treatment did not affect the baseline heart rate values between HTX + BI-9740 and HTX groups (216 ± 13 vs 239 ± 13 beats/min, *p* > 0.05). However, administration of BI-9740 to the recipient rats was associated with a significant shortening in graft re-beating time after reperfusion compared to the placebo group (first beat: 14.6 ± 1.6 s vs. 21.7 ± 2.8 s and regular beating: 29.5 ± 1.8 s vs. 43.7 ± 3.5 s, *p* < 0.05). Additionally, the graft survival rate (i.e. a functioning graft at the time of cardiac function measurement) was 100% in both the HTX and HTX + BI-9740 groups.

### Effect of BI-9740 on graft function after HTX

**Fig. 6:**
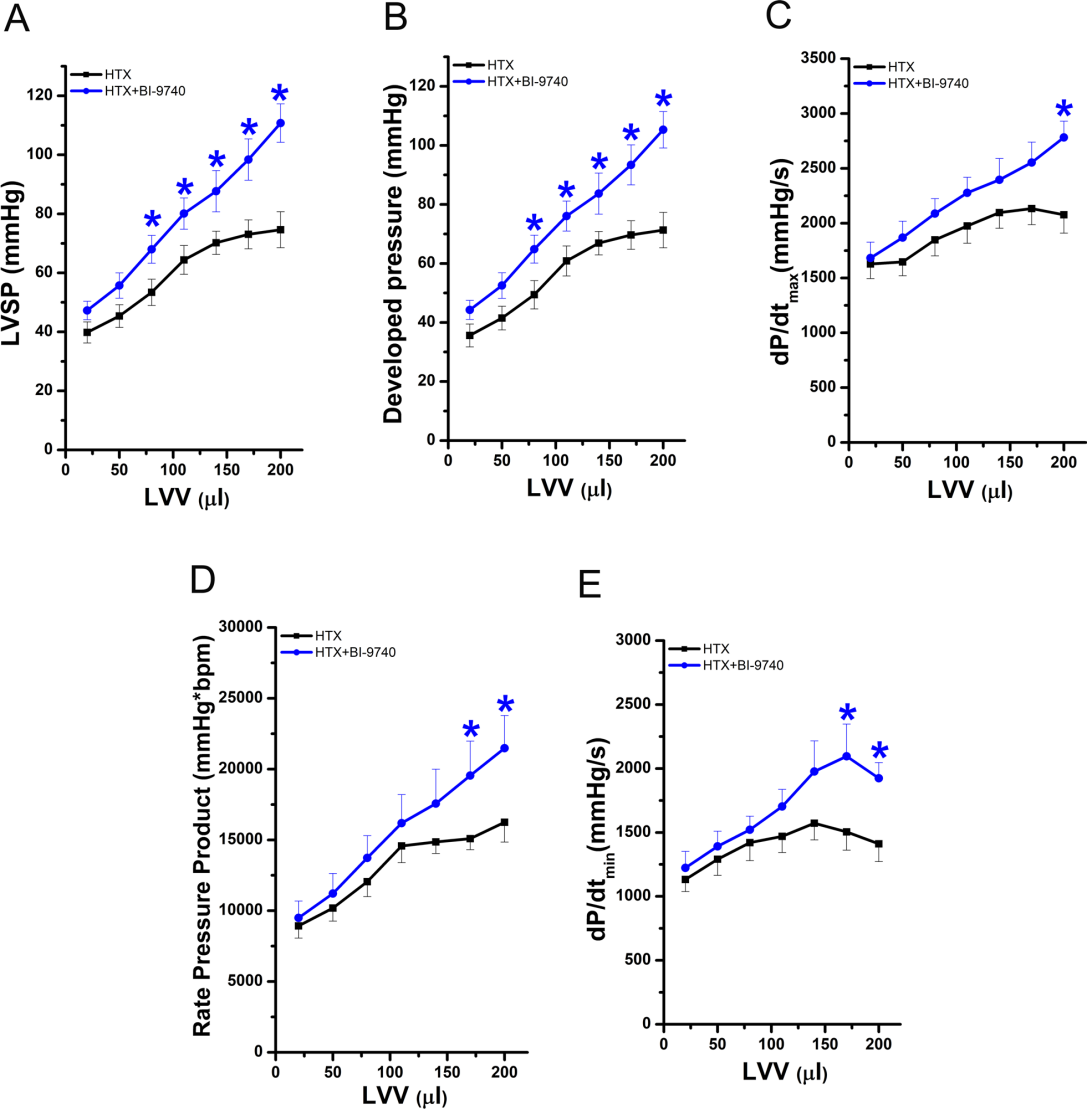
Effect of BI-9740 on left-ventricular (LV) graft function after heart transplantation (HTX). **A** Left-ventricular systolic pressure (LVSP), **B** developed pressure, **C** maximal slope of systolic pressure increment (dP/dt_max_), **D** rate pressure product, and **E** maximal slope of diastolic pressure decrement (dP/dt_min_) were measured at different LV volumes. Values are expressed as mean ± SEM. *p < 0.05 vs. HTX. n = 11–12 rats/group

In the implanted graft, LV post-transplant systolic function was significantly improved, as evidenced by increased LVSP, developed pressure, and dP/dt_max_ (Fig. 6A–C). Moreover, the rate pressure product, a reliable direct indicator of myocardial oxygen demand, was significantly higher in the BI-9740 hearts compared to the placebo group (Fig. 6D). Active myocardial relaxation, evaluated by dP/dt_min_, was also significantly improved with BI-9740 treatment as compared to the placebo group (Fig. 6E).

## Discussion

The purpose of this study was to examine the effects of recipient treatment with a chemical nitrile CatC inhibitor in a rat model of HTX. To the best of our knowledge, this is the first piece of work to suggest that inhibiting CatC with BI-9740 results in diminished major NSP proteolytic activities, ameliorated histopathological changes, reduced neutrophil infiltration, and lowered nitro-oxidative stress in the myocardium with a decrease in the number of the PARP-1-positive cells. Subsequently, this leads to an improved functional recovery of grafts after cardioplegic arrest, followed by ex vivo hypothermic storage and in vivo myocardial blood reperfusion.

Currently, the hearts of brain-dead donors are the major source for HTX. However, brain death is associated with systemic inflammation leading to worse graft outcomes. To distinguish the specific effects of brain death and IR injury, we avoided additional factors that could influence our hypothesis. After short periods of cold ischemia (30 min, 1 h, 1.5 h) reperfusion in rat cardiac allografts results in a significant increase in superoxide production, cardiomyocyte apoptosis, and inflammatory response [21]. Rapid recovery of myocardial function plays a crucial role in determining long-term outcomes because severe hemodynamic changes take place during the early phase following HTX. Therefore, in the present study, the cold ischemia time has been fixed to 1 h and graft function was evaluated 1 h after warm blood reperfusion [17].

Primary graft failure, a frequent complication occurring in the immediate post-operative period, continues to be the main cause of death within the first 30 days after HTX [22]. Given these observations, potential therapeutic strategies are required to protect the grafts by treating the recipients, subsequently improving patient outcomes. The data on the underlying biology associated with primary graft failure, especially the NSP angle, overlaps with the biological profile of CatC inhibitors currently under investigation in the clinic for the inflammatory pulmonary disease non-cystic fibrosis bronchiectasis. Two CatC inhibitors, Brensocatib (Phase 3; ClinicalTrials.gov Identifier: NCT04594369) [23] and BI 1291583 (Phase 2; ClinicalTrials.gov Identifier: NCT05238675), are presently in clinical trials for this indication. In addition, some preclinical results indicate the benefits of CatC inhibition, opening avenues toward drug repurposing strategies. The findings of Jerke et al. using BI-9740 encourage clinical studies involving adjunctive CatC inhibitor administration in patients with proteinase 3-ANCA vasculitis [24]. Xiao et al. showed that the systemic administration of Brensocatib effectively inhibited breast cancer lung metastasis in a mouse model [16]. Chen et al. showed that the small molecule inhibitor SF38 inhibited CatC activity in the bone marrow and blood, decreased the activation of NSPs, and exhibited anti-inflammatory activity in an animal model of acute lung injury [25]. A recent study showed that the downregulation of CatC improved HUVEC viability and enhanced antiapoptotic capacity, anti-oxidative capability, invasive ability, as well as the angiogenic potential of hypoxia/reoxygenation-stimulated human umbilical vein endothelial cells (HUVECs) [26]. This improvement was achieved by repressing the p38 mitogen activated protein kinase (MAPK)/nuclear factor-kappaB (NF-κB) pathway, suggesting a novel promising approach for preeclampsia therapies [26]. In a preclinical murine lung transplant model, the administration of CatC inhibitor to recipients alleviated early primary post-transplant graft dysfunction [27]. These findings together strongly suggest other applications of CatC inhibitors besides non-cystic fibrosis bronchiectasis and make them excellent drug candidates for organ transplantation as well. In our heterotopic transplant rat model, we showed that global IR injury leads to myofiber disorganization, which is related to a dense infiltration of inflammatory cells and interstitial edema. Treatment with BI-9740 resulted in reduced irregular fiber arrangement, decreased inflammatory infiltrates, and less edema. Additionally, the increased number of CatC positive cells in the transplanted heart was reduced by the inhibition of CatC. Administering BI-9740 for 12 days did not induce a loss of body weight, a parameter used for safety assessment, and had no effect on baseline heart rate values compared to rats treated with a placebo.

The over-activation of PARP is involved in various types of tissue injury, including systemic inflammation and IR injury [28]. During IR injury, DNA-strand breaks caused by ROS and reactive nitrogen species lead to PARP-1 activation. Excessive activation of PARP-1 can contribute to tissue injury by driving the cell into an energy crisis [29] and cell dysfunction, and by catalyzing the activation of pro-inflammatory pathways. It is recognized that CatC inhibitors have significant anti-inflammatory properties. MPO is a lysosomal enzyme mainly stored in the azurophilic granules neutrophils. It has been previously shown that pharmacological CatC inhibition does not affect MPO activity [24]. Therefore, MPO can be used as a surrogate marker for neutrophil infiltration. Our data showed that neutrophil infiltration into myocardial tissues, as evidenced by MPO-positive cells, was decreased by BI-9740 in grafts. We previously showed PARP over-activation contributes to reperfusion injury and its pharmacological inhibition prevents PARP activation, improves myocardial contractility/relaxation, coronary blood flow, and endothelial function in the transplanted heart [30]. However, this work did not yield evidence of DNA fragmentation/DNA strand breaks, nor the generation of both superoxide anions and hydrogen peroxide. The absence of ROS generation, as indicated by dihydroethidium fluorescence staining, correlates with a lack of significant protein alterations related to apoptosis, as demonstrated by western blot, and the absence of DNA damage, as evidenced by the TUNEL assay. In the present study, immunohistochemistry revealed an increased expression of nitrotyrosine, an indicator of nitrosative stress, with an increase in the number of PARP-1-positive cells in the transplanted hearts, both of which were reduced by CatC inhibition. Our functional analysis demonstrated that systolic function (evidenced by increased LVSP, developed pressure, and dP/dt_max_) and myocardial relaxation (shown by increased dP/dt_min_) were improved, and rate pressure product was increased by BI-9740 treatment compared to the placebo group.

From a clinical perspective, if an efficient anti-proteolytic therapy up-steam of elastase-related serine proteases by blocking their maturing enzyme CatC [10] could also be demonstrated in experimental models of myocardial global IR injury, this could open a new medical use for available drugs in treating high-risk patients categorized as high priority on the HTX waiting list. Both the 24-week Phase 2 Trial [23] and the ongoing Phase 3 Trial with Brensocatib encourage the use of CatC inhibitors in clinical practice. Importantly, it should be noted that the multiple highly redundant mechanisms of neutrophil defense responses are generally not impaired by CatC inhibitor treatment [9, 31].

In our ongoing research involving pharmacological CatC inhibition in HTX, we focused on questions that could not be addressed in the current model set-up. Firstly, the heterotopic abdominal rat HTX model was selected as appropriate for assessing global IR injury. However, the left ventricle beats in an unloaded condition, leading to a faster recovery following IR injury, making it unsuitable for studying long-term effects. Secondly, the study did not examine the structure and function of the right ventricle. Thirdly, the experiment used an isoimmune model to explore IR independently from acute rejection, so the potential adverse effects of alloimmune reactions resulting from HTX were not investigated. Fourth, only one dose of 20 mg/kg was tested. Lastly, while the measurement of graft function study did not incorporate a non-ischemic heart transplant group, such a group was, however, included in the immunohistochemistry and western blot experiments. Despite these limitations, CatC inhibitors offer great promise for the future heart surgery patients.

## Conclusion

Pharmacological inhibition of CatC decreases NE and CatG activities, ameliorates histopathological changes, reduces myocardial neutrophil infiltration and nitro-oxidative stress with a decrease in the number of PARP-1-positive cells. Consequently, it results in an improvement in the LV graft function after HTX in rats. The clinical relevance of our findings should be further carefully evaluated.

### Supplementary Information

**Additional file 1: Figure S1.** Effects of BI-9740 (BI) on reactive oxygen species (ROS) generation and DNA strand breaks in grafts after transplantation (HTX). (A) Representative images of dihydroethidium fluorescence staining (× 10, scale bar: 1 mm) followed by semi-quantitative analysis. Dihydroethidium is freely permeable to cell membranes and emits a red fluorescent signal when oxidized by ROS to ethidium. (1 picture from 10 rats/group). Values are expressed as mean ± SEM. (B) Representative images of myocardial tissue showing pink nuclei with fragmented DNA, visualized by terminal deoxynucleotidyl transferase-mediated dUTP nick end-labeling (TUNEL) staining. Blue nuclei represents 4’,6-diamino-2-phenylindole staining (magnification × 40; scale bar: 100 µm) followed by quantification based TUNEL-positive cells expressed as a percentage over the total cell count. The evaluation of TUNEL assay was carried out on 6 randomly selected non-overlapping fields within each rat’s myocardial tissue (59–60 pictures from 10 rats/group). Values are expressed as mean ± SEM and correspond to the mean of all pictures. The samples were assessed by examiners who were blinded to the experimental groups.

## Data Availability

“The datasets generated and/or analyzed during the current study are available from the corresponding author on reasonable request.”

## Abbreviations

BCA: Bicinchoninic acid
BSA: Bovine serum albumin
CatC: Cathepsin C
CatG: Cathepsin G
dP/dt_max_: Maximum rate of rise of left-ventricular pressure
dP/dt_min_: Maximum rate of fall of left-ventricular pressure
DPPI: Dipeptidylpeptidase I
GAPDG: Glyceraldehyde-3-phosphate dehydrogenase
HTX: Heart transplantation
HUVECs: Human umbilical vein endothelial cells
IR: Ischemia/reperfusion injury
LV: Left-ventricular
LVSP: Left-ventricular systolic pressure
MAPK: Mitogen activated protein kinase
MPO: Myeloperoxidase
NE: Neutrophil elastase
NF-κB: Nuclear factor-kappaB
NSPs: Neutrophil serine proteases
PARP: Poly(ADP-ribose) polymerase
PBS: Phosphate-buffered saline
RIPA: Radioimmunoprecipitation assay
ROS: Reactive oxygen species
SDS: Sodium dodecyl sulfate
SEM: Standard error of the mean
TBS: Tris-buffered saline
TUNEL: Terminal deoxynucleotidyl transferase-mediated dUTP nick-end labeling
