# Supplementary figures and images for: Pharmacological inhibition of the cysteine protease cathepsin C improves graft function after heart transplantation in rats
Source: J Transl Med. 2023 Nov 9;21:799. doi: 10.1186/s12967-023-04659-6 (PMC10636924; doi:10.1186/s12967-023-04659-6)

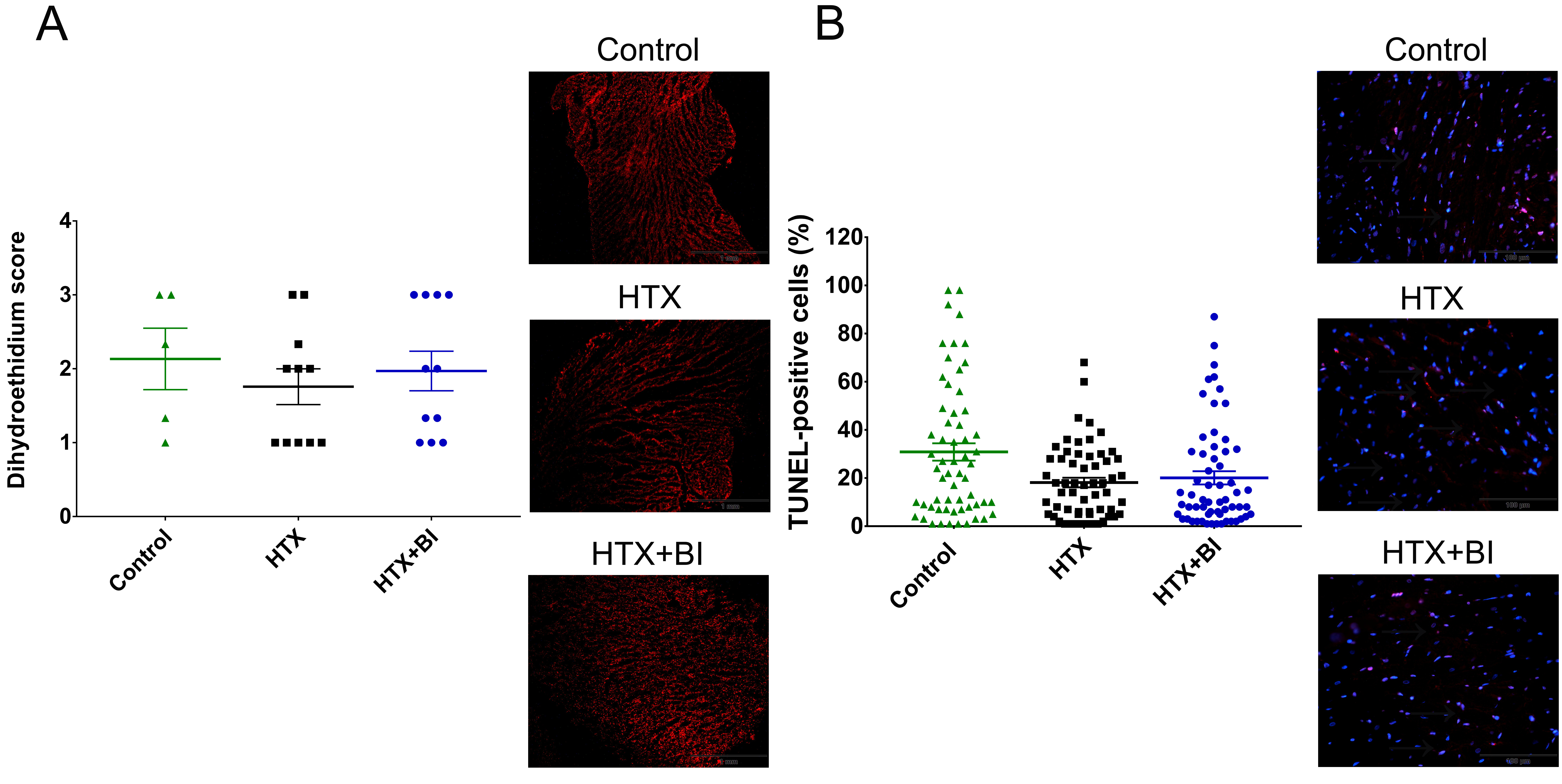

Supplement: Supplementary file 1 — Additional file 1: Figure S1. Effects of BI-9740 (BI) on reactive oxygen species (ROS) generation and DNA strand breaks in grafts after transplantation (HTX). (A) Representative images of dihydroethidium fluorescence staining (× 10, scale bar: 1 mm) followed by semi-quantitative analysis. Dihydroethidium is freely permeable to cell membranes and emits a red fluorescent signal when oxidized by ROS to ethidium. (1 picture from 10 rats/group). Values are expressed as mean ± SEM. (B) Representative images of myocardial tissue showing pink nuclei with fragmented DNA, visualized by terminal deoxynucleotidyl transferase-mediated dUTP nick end-labeling (TUNEL) staining. Blue nuclei represents 4’,6-diamino-2-phenylindole staining (magnification × 40; scale bar: 100 µm) followed by quantification based TUNEL-positive cells expressed as a percentage over the total cell count. The evaluation of TUNEL assay was carried out on 6 randomly selected non-overlapping fields within each rat’s myocardial tissue (59–60 pictures from 10 rats/group). Values are expressed as mean ± SEM and correspond to the mean of all pictures. The samples were assessed by examiners who were blinded to the experimental groups. [file 12967_2023_4659_MOESM1_ESM.jpg]
